# Correction: Ascorbic acid/Fe^0^ composites as an effective persulfate activator for improving the degradation of rhodamine B

**DOI:** 10.1039/c8ra90046a

**Published:** 2018-06-04

**Authors:** Xiangyu Wang, Yi Du, Huiling Liu, Jun Ma

**Affiliations:** Faculty of Environmental Science and Engineering, Kunming University of Science and Technology Kunming 650500 P. R. China imusthlee2014@sina.com; School of Municipal and Environmental Engineering, State Key Laboratory of Urban Water Resources and Environment, Harbin Institute of Technology Harbin 150090 P. R. China

## Abstract

Correction for ‘Ascorbic acid/Fe^0^ composites as an effective persulfate activator for improving the degradation of rhodamine B’ by Xiangyu Wang *et al.*, *RSC Adv.*, 2018, **8**, 12791–12798.

The authors regret that the unit on the *x*-axis of Fig. 1 was incorrectly written as “% wt” rather than “‰ wt” in the original article. The correct version of Fig. 1 is presented below.

**Fig. 1:**
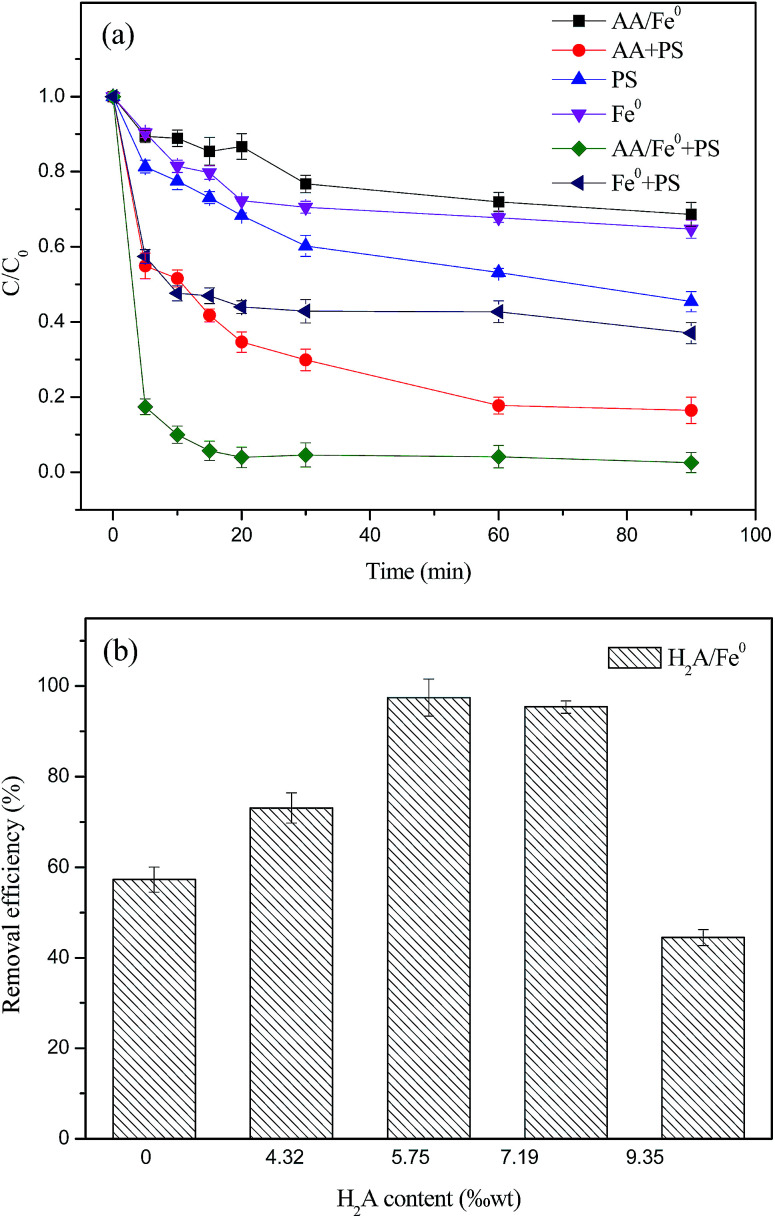
(a) Comparison of removal efficiency of RhB in different systems (*C*_0_ = 50 mg L^−1^, PS dosage = 1.4 g L^−1^, Fe^0^ dosage = 1 g L^−1^, H_2_A/Fe^0^ dosage = 1 g L^−1^, H_2_A dosage = 1.6 g L^−1^ and *T* = 298 K); (b) effect of H_2_A concentration on removal efficiency of RhB in the H_2_A/Fe^0^–PS system (*C*_0_ = 50 mg L^−1^, Fe^0^ dosage = 0.8 g L^−1^, *T* = 298 K and the solution volume is 50 mL).

The Royal Society of Chemistry apologises for these errors and any consequent inconvenience to authors and readers.

## Supplementary Material

